# Pathogenesis of swine influenza virus (Thai isolates) in weanling pigs: an experimental trial

**DOI:** 10.1186/1743-422X-6-34

**Published:** 2009-03-25

**Authors:** Donruethai Sreta, Roongtham Kedkovid, Sophon Tuamsang, Pravina Kitikoon, Roongroje Thanawongnuwech

**Affiliations:** 1 Chulalongkorn University, Bangkok, Thailand; 2 National Institute of Animal Health, Bangkok, Thailand

## Abstract

**Background:**

The objective of this study is to investigate the pathogenesis of swine influenza virus (SIV) subtype H1N1 and H3N2 (Thai isolates) in 22-day-old SPF pigs.

**Results:**

The study found that all pigs in the infected groups developed typical signs of flu-like symptoms on 1–4 days post- infection (dpi). The H1N1-infected pigs had greater lung lesion scores than those of the H3N2-infected pigs. Histopathological lesions related to swine influenza-induced lesions consisting of epithelial cells damage, airway plugging and peribronchial and perivascular mononuclear cell infiltration were present in both infected groups. Immunofluorescence and immunohistochemistry using nucleoprotein specific monoclonal antibodies revealed positive staining cells in lung sections of both infected groups at 2 and 4 dpi. Virus shedding was detected at 2 dpi from both infected groups as demonstrated by RT-PCR and virus isolation.

**Conclusion:**

The results demonstrated that both SIV subtypes were able to induce flu-like symptoms and lung lesions in weanling pigs. However the severity of the diseases with regards to lung lesions both gross and microscopic lesions was greater in the H1N1-infected pigs. Based on phylogenetic analysis, haemagglutinin gene of subtype H1N1 from Thailand clustered with the classical H1 SIV sequences and neuraminidase gene clustered with virus of avian origin, whereas, both genes of H3N2 subtype clustered with H3N2 human-like SIV from the 1970s.

## Background

Swine influenza is an acute, highly contagious, respiratory disease caused by type A influenza virus infection. Currently, 16 haemagglutinin (HA) subtypes and 9 neuraminidase (NA) subtypes are identified. Three main subtypes currently circulating in the pig population are classical swine influenza virus (SIV) and reassortant viruses of H1N1, H3N2 and H1N2 [1]. However, pigs can also be infected with other subtypes of influenza A viruses. Pig plays a substantially important role in the ecology of influenza A virus [2] since they can act as a 'mixing vessel'. When co-infections among human, avian or swine influenza viruses occur within a specific host, any new subtype can be produced by antigenic reassortment [3].

Normally, SIV infects the epithelial lining of the respiratory tract producing clinical signs consisting of cough, fever, lethargy and anorexia. SIV-associated gross lung lesions observed in pigs are characterized by multifocal well-demarcated purplish-red lesions in the cranioventral areas of lung lobes known as a checker-board lung. SIV-induced microscopic lesions consist of epithelial disruption and attenuation in the bronchioles with later found hyperplastic proliferation and bronchiolitis obliterans. Mild to moderate peribronchiolar and perivascular lymphocytic infiltration occurs at nearly all levels of the airways. Viral antigen can be detected in epithelial cells of airways by immunohistochemistry (IHC) staining [4].

In Thailand, H1N1 SIV was the first subtype isolated from pigs with an influenza-like symptom in 1990 [5]. Currently, both H1N1 and H3N2 subtypes are commonly found among the pig population in the country according to serological studies and virus isolation [6]. Subsequently, in 2005 a new subtype H1N2 was isolated from pigs in Saraburi province [6]. Wang et al. [7] reported that the H1 HA antigen was more resistant to natural cleavage into its two subunits (HA1 and HA2 subunits) than H3 HA antigen. It is possible that H3 virus could easily bind to the specific receptors resulting in better ability to infect cells than H1 virus. Moreover, human H3N2 virus could induce higher antibody response than that of H1N1 virus as revealed by hemagglutination-inhibition (HI) titers [8]. In addition, Van Reeth et al. [9] demonstrated that pigs infected with a European H3N2 virus induced higher HI titers compared to a European H1N1 virus.

In Thailand, pathogenesis of SIV subtype H1N1 and H3N2 infection in swine has never been studied. Since different subtypes of the influenza type A viruses isolated from pigs are found to cause different pathogenic levels in pigs, the objective of this study is to investigate the pathogenesis of SIV (Thai isolates) subtype H1N1 (A/swine/Thailand/HF6/05) and H3N2 (A/swine/Thailand/S1/05) in weanling SPF pigs. Genetic characterization of the HA gene of both studied viruses were also performed in this report.

## Results

### Clinical evaluation

All pigs in the SIV infected groups showed clinical respiratory signs such as nasal discharge, coughing, sneezing and conjunctivitis by 1–4 dpi with mean clinical scores from 1.5 to 2.0. However, there were no significant differences between the infected groups. The negative control group showed no clinical respiratory signs. All studied pigs had no fever (≤ 40°C).

### Pathological evaluation

At necropsy, lung macroscopic lesions characterized by dark plum-colored, consolidated areas on lung lobes were observed in both-infected pigs. Grossly, the lung lesions were seen mostly in the cranioventral areas. Percentages of gross lung lesions are shown in Table 1. The lung lesions were severe the most at 2 dpi, especially in the H1N1-infected pigs. The lungs of all control pigs had no macroscopic lesions at all necropsied dates.

**Table 1:** Percentages of gross lung lesions and the presence of SIV-specific antigen based on immunohistochemistry (IHC) staining

	mock*	H1N1**		H3N2**	
	
dpi	Pig 1 (%)	Pig 1 (%)	Pig 2 (%)	Pig 1 (%)	Pig 2 (%)
2	0.0(-)	36.0(+)	33.0(+)	20.0(+)	2.0(+)
4	0.0(-)	5.0(+)	3.0(+)	2.0(+)	1.0(-)
12	0.0(-)	6.0(+)	0.5(-)	0.0(-)	0.0(-)

*n = 1, **n = 2.

dpi = days post infection

(-) negative antigen detection by IHC, (+) positive antigen detection by IHC

Microscopic lung lesions in both infected groups showed a thin layer bronchiolar lining of attenuated epithelium. Subsequently, necrosis and sloughing off of the epithelial cells with loose lymphocytes infiltrating around the bronchiole was evident. The microscopic lung lesions were scored 2, 1 and 0 in both infected groups at 2, 4 and 12 dpi, respectively. The lungs of all control pigs had no microscopic lesion scores at any necropsied date.

**Figure 1:**
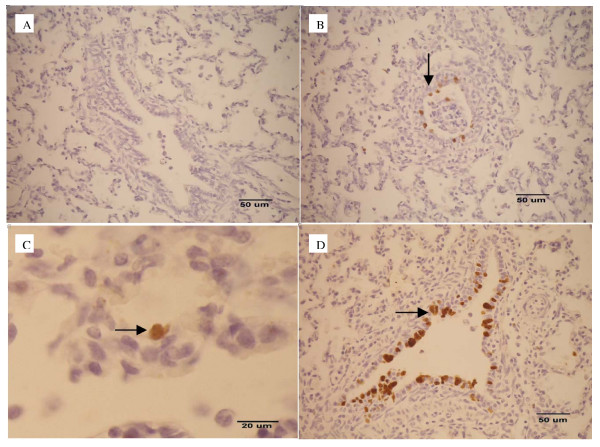
SIV antigen staining by IHC, (A) negative control, (B) dark brown staining cells (arrow) of the SIV-positive control, (C) SIV-positive staining on alveolar epithelial cells and (D) bronchiolar epithelial cells.

SIV antigen was observed in the lung tissues by both IFA and IHC (Table 1). IFA using nucleoprotein specific monoclonal antibodies was done at the necropsied dates revealing positive apple-green color in the nuclei of the alveolar, bronchial and bronchiolar epithelial cells in all infected pigs as early as 2 dpi. Later, IHC also demonstrated strong positive dark brown staining in the nuclei of those lung cells similar to the IFA which were correlated well with the lung lesions. However, IHC positive staining cells were also found in macrophages located mainly in the lung lesion at the cranioventral areas (Figure 1).

### Virus isolation

There were no differences in the virus titers in both experimental SIV-infected groups. The virus titers at 2 dpi were between 10^2 ^– 10^3 ^TCID_50_/ml either from lung tissues or from bronchoalveolar lavage fluid (BALF) in both infected groups. There was no virus titre at 4 or 12 dpi neither from lung nor from BALF in both infected groups or in the control pigs at any necropsied date (data not shown).

### RT-PCR

RT-PCR using specific primers for the matrix protein gene to confirm the presence of SIV in the nasal swabs revealed positive results only between 2–4 dpi in the H1N1-infected group and only at 2 dpi in the H3N2-infected group. SIV genetic material was not found in the nasal swabs or the sera of the negative control pigs.

### Haemagglutination-inhibition (HI) assay

Sera from pigs in the control group had no antibody titres (≤ 1:10 HI titre) to either H1N1 or H3N2 subtypes at all collected dates. Sera from pigs in the H3N2-infected group had no antibody titres (≤ 1:10 HI titre) to the H1N1 subtype. Similarly, sera from pigs in the H1N1-infected group had no antibody titres (≤ 1:10 HI titre) to the H3N2 subtype. H3N2-infected pigs had 1:40 HI titre at 4 and 12 dpi to the H3N2 subtype. Interestingly, H1N1-infected group had the HI titre (1:160 HI titre) to the H1N1 subtype at 12 dpi.

### Pathogenic bacterial culture and identification

Pathogenic bacterial culture yielded negative results from the tracheal swabs and lung tissues in all groups at 2, 4 and 12 dpi. In addition, *Mycoplasma hyopneumoniae *were not found by PCR tested from lung tissues in all pigs.

### DNA Sequencing and Phylogenetic analysis

The nucleotide sequences in this study contained 1700 bp (36–1736) HA gene of H1N1, 1686 bp (37–1723) HA gene of H3N2 and 1409 bp NA gene of both subtypes from the selected Thai isolates. The HA and NA gene nucleotide sequences of the Thai isolates of both subtypes from Chonburi province in this study were analyzed by BLAST (Basic Local Alignment Search Tool) program available at  demonstrating that both HA and NA sequences of the H1N1 virus had the highest homology to A/swine/Chonburi/05CB1/2005 (H1N1) and the H3N2 virus had the highest homology to A/swine/Chonburi/05CB2/2005 (H3N2) (Table 2). Geographic influence definitely played a major role in those studied viruses.

**Table 2:** Subtype and homology analysis of HA and NA genes of the H1N1 and H3N2 challenge viruses

Virus	Subtype	Virus with homology of HA gene	%*	Virus with homology of NA gene	%*
A/swine/Thailand/HF6/05	H1N1	A/swine/Chonburi/05CB1/05 (H1N1)	100	A/swine/Chonburi/05CB1/05 (H1N1)	100
		A/swine/Chonburi/06CB2/06 (H1N1)	99	A/swine/Chonburi/06CB2/06 (H1N1)	99
		A/Thailand/271/05 (H1N1)	97	A/Thailand/271/05 (H1N1)	97
		A/swine/Tennessee/4/78 (H1N1)	91	A/Swine/England/195852/92 (H1N1)	93
		A/swine/Tennessee/2/78 (H1N1)	91	A/swine/Cotes d'Armor/1488/99 (H1N1)	93
					
A/swine/Thailand/S1/05	H3N2	A/swine/Chonburi/05CB2/05 (H3N2)	99	A/swine/Chonburi/05CB2/05 (H3N2)	99
		A/swine/Nakhon pathom/NIAH586-1/05 (H3N2)	97	A/swine/Nakhon pathom/NIAH586-1/05 (H3N2)	97
		A/swine/Nakhon pathom/NIAH586-2/05 (H3N2)	96	A/swine/Nakhon pathom/NIAH586-2/05 (H3N2)	96
		A/swine/Chachoengsao/NIAH586/05 (H3N2)	98	A/swine/Chachoengsao/NIAH586/05 (H3N2)	95
		A/Albany/20/74(H3N2)	88	A/Albany/20/74 (H3N2)	90

* Percent homology of nucleotide sequence from BLAST analysis

**Figure 2:**
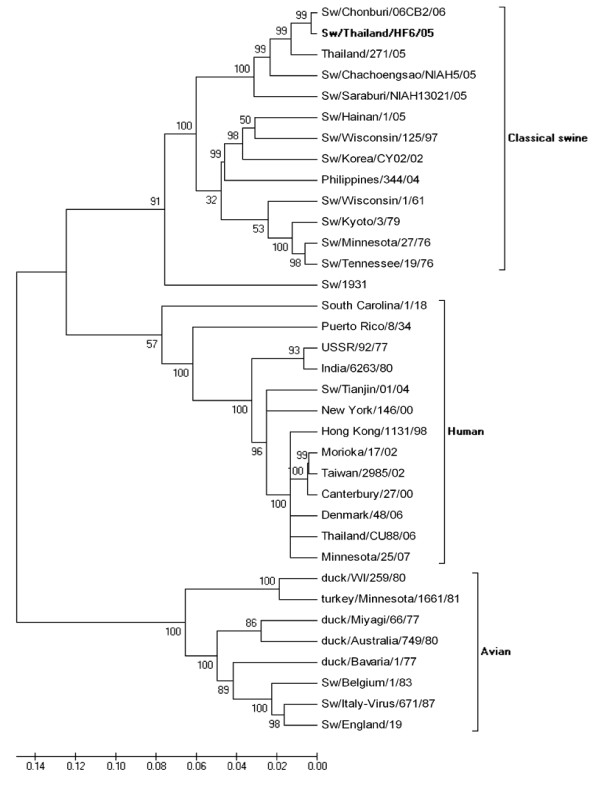
**Phylogenetic tree of the HA [GenBank:**FJ688566] **gene of 1700 bp (36–1736) H1 influenza A viruses**.

**Figure 3:**
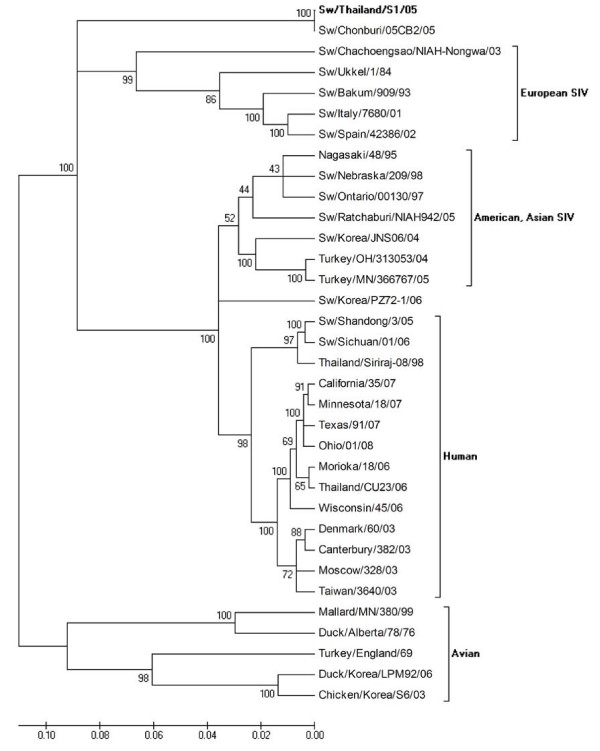
**Phylogenetic tree of the HA [GenBank:**FJ688268] **gene of 1686 bp (37–1723) H3 influenza A viruses**.

**Figure 4:**
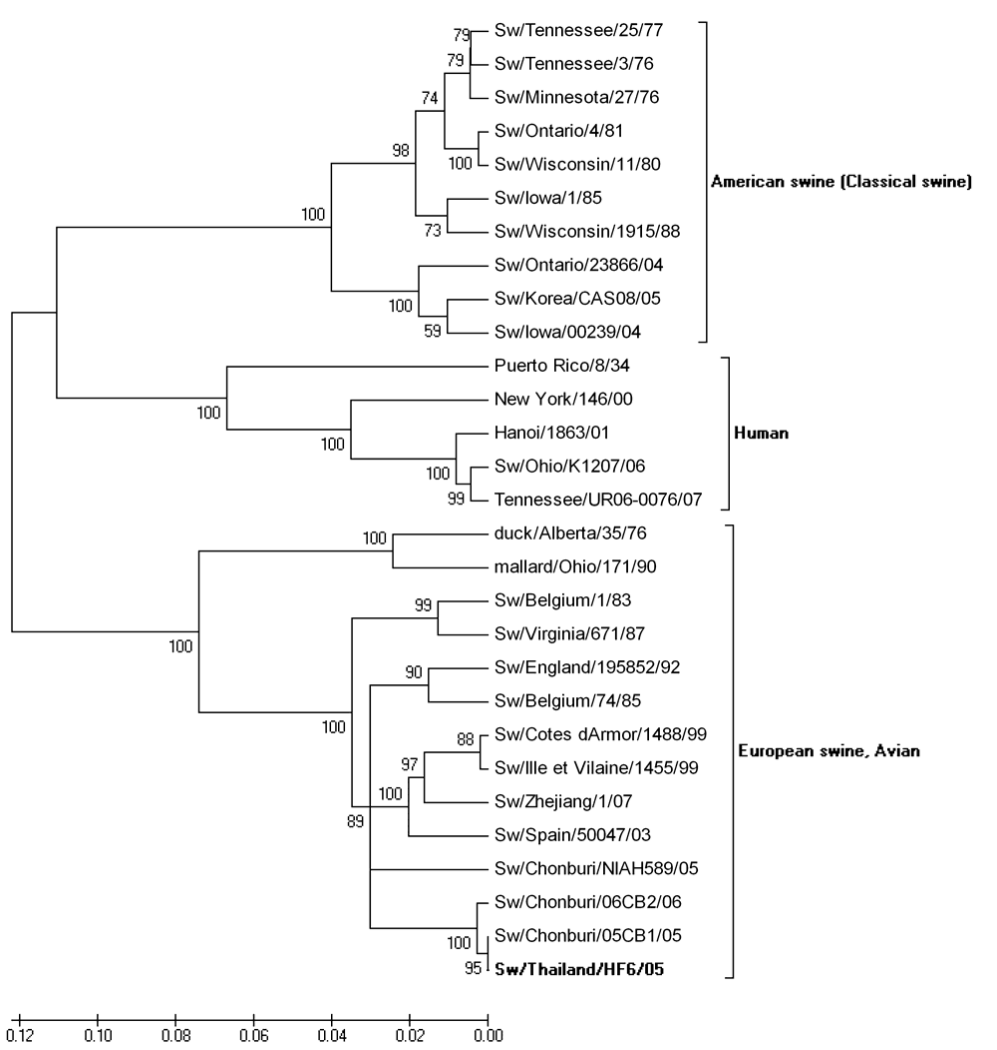
**Phylogenetic tree of the NA [GenBank:**FJ688267] **gene of 1409 bp N1 influenza A viruses**.

**Figure 5:**
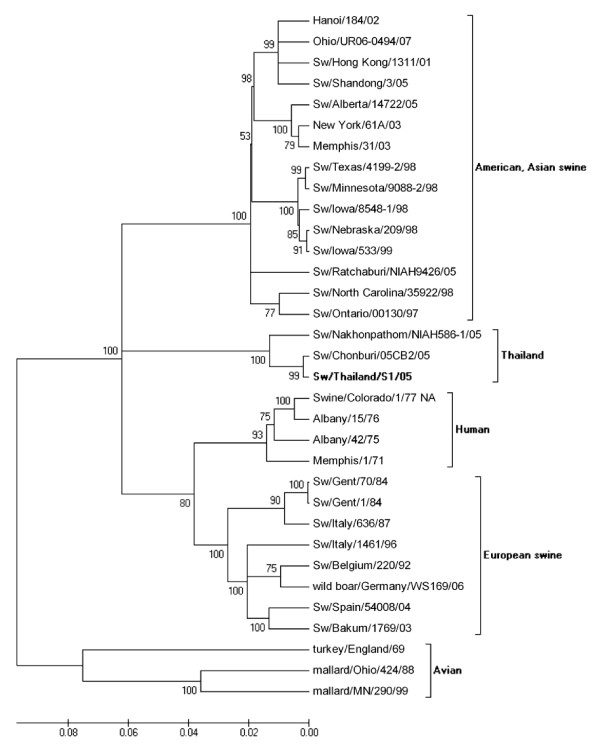
**Phylogenetic tree of the NA [GenBank:**FJ688269] **gene of 1409 bp N2 influenza A viruses**.

Phylogenetic analysis of the HA gene from the selected isolates revealed that nucleotide sequences of H1 viruses had three major clusters, classical swine, avian and human influenza virus (Figure 2) and nucleotide sequences of H3 viruses had four major clusters, European, American and Asian swine, avian and human influenza viruses (Figure 3). Phylogenetic analysis of the NA gene from the selected isolates revealed that nucleotide sequences of N1 viruses contained three clusters, American swine (classical swine), Human, and European swine and avian influenza virus as shown in Figure 4. The nucleotide sequences of N2 viruses from the selected isolates had three clusters, American and Asian swine, European swine and human and avian influenza virus as shown in Figure 5. The results showed that the nucleotide sequences of the HA [GenBank:FJ688266] gene of A/Thailand/HF6/05 (H1N1) belonged to the classical swine H1 lineage and the NA [GenBank:FJ688267] gene belonged to avian N1 lineage, and both HA [GenBank:FJ688268] and NA [GenBank:FJ688269] genes of A/Thailand/S1/05 (H3N2) belonged to the human H3N2 lineage.

## Discussion

The results demonstrated that both SIV subtypes (Thai isolates) were able to induce the flu-like symptoms and lesions compatible with viral pneumonia in the cranioventral areas and were able to cause broncho-interstitial pneumonia similar to previous reports [4,10-12]. The pig lung is certainly the major site of swine influenza virus replication [1] since we did not find any viraemic pig or SIV antigen detection outside the lung tissue at any day of infection. IHC and RT-PCR seemed to be more sensitive than the virus isolation. The course of infection was limited to less than a week in both SIV-infected groups as SIV antigen detection was found positive only at 2–4 dpi. The SIV antigen was found in the nuclei of the bronchial and bronchiolar epithelial cells, pneumocytes and pulmonary macrophages with similar levels in both SIV-infected groups indicating no differences between the two subtypes in the viral protein production or replication. It should be noted that both studied Thai isolates of both subtypes replicated only in the respiratory tract of pigs and shed the virus in the nasal secretions similar to other SIV. The clinical respiratory signs and lung pathology in swine influenza-infected pigs are commonly induced by the pro-inflammatory cytokines such as IFN-α, TNF-α, IL-1 α and β, and IL-6 in bronchoalveolar lavage fluids and the amount of viral load in the lung tissue [13]. Our results showed that pigs in both SIV-infected groups showed more severe clinical respiratory signs and had higher body temperature compared to the control pigs. Although, none of the studied pigs had fever, pigs in both SIV-infected groups had slight elevated body temperature. However, cytokines responsible for those clinical signs and lesions were not done in this study.

It is generally believed that there is no cross-protection between H1N1 and H3N2 viruses [9]. The HI test between H1N1 and H3N2-infected groups had no cross HI antibody reaction in this study. Commonly, infection with H3N2 viruses induces higher HI titers than those of H1N1 infection [9,14]. Wang et al. [7] found that the cleavage site of HA antigen of H3 virus (A/Panama/2007/99) was cleaved easier than that of H1 virus (A/New Caledonia/20/99) in transiently transfected 293T cells possibly making the H3 viruses more immunogenic. Similarly, a recombinant vaccine study in elderly humans using A/Panama/2007/99 (H3N2), A/New Caledonia/20/99 (H1N1) or influenza virus type B as virus sources showed that H3N2 virus induced significant higher HI titres than that of H1N1 virus [15]. In contrast, the H1N1-infected pigs in this study induced higher HI titres than that of the H3N2-infected pigs at 12 dpi. Similarly, Kitikoon et al. [14] showed that HI titre of the H1N1-infected pigs at 7 dpi was higher than that of the H3N2-infected pigs. However, the HI titres at 14 and 21 dpi in the H3N2 virus-infected pigs were higher in that study. Different viruses used in the studies may yield different results due to the origin of those isolates. Interestingly, the antigenic site of the H1N1 used in this study contained the changed in the amino acid sequence of haemagglutinin which may result in increased immunogenicity and induced more severe clinical diseases. However, the antigenic site of the H3N2 virus used in this study had no amino acid changes. In addition, we did not have the HI results after 12 dpi in our study.

Recently, genetic analysis of the HA gene of Thai SIV [16] showed that the recent Thai H1N1 virus belonged to the classical swine H1N1 lineage and had the highest homology to the human isolate A/Thailand/271/05(H1N1). The H3N2 subtype belonged to the human H3N2 lineage and showed highest homology to the H3N2 human influenza virus from the 1970s A/Bilthoven/2600/75(H3N2) [16]. Similarly, in our study nucleotide sequences of both HA and NA genes of A/Thailand/HF6/05(H1N1) also belonged to the classical swine H1N1 lineage and A/Thailand/S1/05(H3N2) belonged to the human H3N2 lineage. Since those viruses were isolated from the same province and in the same year, geographic distribution could be an explanation for the similarity of those viruses.

Interestingly, both viruses in this study were isolated from the same farm in August 2005. We did not find any genetic shift in those viruses or any reassortant virus such as H1N2 subtype occurring in the farm (unpublished data). Whole genome sequencing of all 8 genes is needed to be evaluated in both viruses. It is possible that the new reassortant virus might occur in Thailand since both studied viruses were isolated from the same farm. However, our results showed that the Thai SIV subtypes, H1N1 and H3N2, are still circulating in the pig population in Thailand since the first report in 1990. Our studied viruses were able to induce flu-like lesions in weanling pigs. Surveillance and molecular investigation of SIV in the country should be done continuously to prevent the future emerging or re-emerging influenza A virus in pigs population.

## Conclusion

The results of this study may assist in the prevention and control of SIV infection in Thailand, especially for H1N1 and H3N2 subtypes. Based on the percentages of cranioventral pneumonic lesion and times of virus shedding, the H1N1 virus might play a major role in respiratory diseases in weanling pigs in that farm. More works are needed in the co-infection model with other respiratory pathogens and in the prevention and control of the SIV-related diseases in Thailand. In this study, investigations on virus transmissibility between sentinel animals housing together with infected animals were not performed. Therefore, whether these Thai H1N1 and H3N2 subtypes will be transmitted efficiently in the field situation requires further experimental and epidemiologic studies.

## Methods

### Viruses

Swine influenza virus subtype H1N1 (A/swine/Thailand/HF6/05) and H3N2 (A/swine/Thailand/S1/05) were isolated from weanling pigs with respiratory signs in the same farm in Chonburi province in 2005. The viruses were propagated in embryonated chicken eggs for 3 passages and the virus concentration (10^7 ^TCID_50_/ml) was evaluated in Madin-Darby canine kidney (MDCK) cells and stored at -80°C until used.

Experimental DesignAnimal usage and handling protocols were approved by Chulalongkorn University-Faculty of Veterinary Science Animal Care and Use Committee (protocol No. 55/2549). The animals were housed at the BSL2 animal facility of the National Institute of Animal Health, Bangkok, Thailand throughout the experiment. Fifteen 22-day-old crossbred SPF pigs serologically negative for porcine reproductive and respiratory syndrome virus (PRRSV), Pseudorabies virus (PRV), *Mycoplasma hyopneumoniae *and SIV were obtained from a commercial farm and assigned to 3 different groups. The infected groups contained six pigs each and were intratracheally inoculated with H1N1 and H3N2 viruses (5 ml of 10^7 ^TCID_50_/ml) in group 1 and 2, respectively. Group 3 served as a negative control group containing three pigs which were mockedly infected with 5 ml of media (minimal essential medium (MEM)). Two pigs from the infected groups and one pig from the control group were necropsied at 2, 4 and 12 days post-infection (dpi). Nasal swabs were collected at 0, 1, 2, 3, 4, 5, 7, 10 and 12 dpi. Following collection, nasal swabs were immediately placed in the infected MEM with 5% BSA, 300 U/ml penicillin, 300 μg/ml streptomycin and 1–2 μg/ml trypsin) and stored at -80°C for evaluation of virus shedding. At each necropsy, sera were collected for assessing antibody response, bronchoalveolar lavage fluid (BALF) were collected for virus isolation and tissue samples from all organs were collected for microscopic lesion detection. In addition, tracheal swabs were tested for bacterial culture and identification.

### Clinical evaluation

Pigs were evaluated daily for respiratory disease symptoms including coughing, sneezing, tachypnea, dyspnea, nasal discharge and conjunctivitis. Pigs were observed and scored for the respiratory signs (ranged from 0 to 5) as previously described [12]. Rectal temperature was also measured daily. Fever was recorded when the rectal temperature ≥ 40°C.

### Pathological evaluation

At necropsy, significant macroscopic lesions of all organs and percentage of lung lesions were recorded. Tissue samples from all lung lobes were collected and immediately identified for the presence of SIV-antigen by immunofluorescence assay (IFA) using anti-influenza A nucleoprotein monoclonal antibody (HB654404 B.V. EUROPEAN VETERINARY LABORATORY, the Netherlands).

Tissue samples from all organs were fixed in 10% neutral buffered-formalin, processed and embedded in paraffin as described previously [4]. Sections were cut approximately 4–6 μm thick for histopathological and immunohistochemistry (IHC) tests using anti-influenza A nucleoprotein monoclonal antibody as previously described [17]. Microscopic lesions were scored as previously described [18] and the scores were ranged from 0 to 3; 0 = normal bronchioles, 1 = mild bronchiolitis, 2 = moderate bronchiolitis, 3 = severe bronchiolitis.

### Virus isolation

SIV was isolated from nasal swabs, sera, BALF and lung tissues according to Kitikoon et al. [12] and the titres (TCID_50_/ml) were calculated according to Reed and Muench [19]. Briefly, ten-fold serial dilutions of the nasal swab solution were inoculated onto MDCK cells and incubated until the cytopathic effect (CPE) was observed for at least 3 cell culture passages. Virus was identified by influenza A virus-specific staining. Prior to staining, cells were fixed with 4% phosphate-buffered formalin and washed with 0.5% Tween-20 in PBS (washing solution). Subsequently, the cells were incubated for 1 h with anti-influenza A nucleoprotein monoclonal antibody diluted 1:1,000 in the washing solution containing 1% BSA (diluting solution). After washing, the cells were incubated 1 h with the rabbit anti-mouse IgG conjugated horseradish peroxidase (Dako Cytomation, Carpinteria, California) diluted 1:400 with the diluting solution. The color was developed using a chromogen aminoethyl carbazole substrate (Sigma, St. Louis, Missouri). Each procedure contained mock-infected negative control cells and positive control cells infected with a known-titreed virus.

### RT-PCR

Sera and nasal swabs were performed to evaluate viraemia and virus shedding using M-gene specific RT-PCR as previously described [10]. Viral RNAs were extracted using QIAamp Viral RNA Mini Kit (Qiagen, Valencia, CA) from 200 μl volume of each nasal swabs and sera. The RT-PCR was performed using Promega One step RT-PCR (Promega, USA) containing a specially formulated enzyme blend for both reverse transcription and PCR. The forward and reward primers were 5' TGA TCT TCT TGA AAA TTT GCA G 3'and 5' TGT TGA CAA AAT GAC CAT CG 3', respectively [20]. The expected amplicon size is 276 bp. The RT-PCR was run at 94°C for 3 min for reverse transcription followed by 30 cycles of denaturing at 94°C for 30 s, annealing at 55°C for 30 s and extension at 72°C for 45 s and ended with a final extension step at 72°C for 7 min. The amplified PCR products were analyzed on a 1.5% agarose gel electrophoresis.

### Haemagglutination-inhibition (HI) assay

Sera were evaluated for the HI antibody titres to both of the SIV subtypes, H1N1 and H3N2 using 0.5% chicken erythrocytes for haemagglutination. All sera were absorbed with Trypsin-Heat-Periodate to reduce nonspecific inhibitors before HI testing [3]. Virus antigens utilized in the HI assays were the challenge viruses, H1N1 (A/swine/Thailand/HF6/05) and H3N2 (A/swine/Thailand/S1/05).

### DNA Sequencing

Full-length HA and NA genes of the studied subtype H1N1 and H3N2 were amplified using universal and specific primers [21,22] with some modifications. The RT-PCR products were analyzed by 1% agarose gel electrophoresis, purified by NucleoSpin Extract II (Macherey-Nagel, Düren, Germany) and cloned into pGEMT Easy (Promega, Madison, WI, USA). Plasmids containing the viral genes were purified by NucleoSpin Plasmid (Macherey-Nagel, Düren, Germany) and sequenced by using synthetic oligonucleotides. Sequence data were edited and analyzed using Bioedit software. The phylogenetic trees were conducted in MEGA4 [23] using neighbor-joining method with 1000 times bootstrapping replicates [24].

## Competing interests

The authors declare that they have no competing interests.

## Authors' contributions

DS carried out the virology, pathology, molecular genetic studies and animal experiment, analyzing data and drafting the manuscript, RK helped in animal work and molecular work, ST helped animal work and pathology, PK virology and revising the manuscript, RT experimental design, pathology and drafting the manuscript and final approval. All authors have read and approved the final manuscript.
